# Traumatic Dental Injuries, Treatment, and Complications in Children and Adolescents: A Register-Based Study

**DOI:** 10.1055/s-0041-1723066

**Published:** 2021-02-03

**Authors:** Austė Antipovienė, Julija Narbutaitė, Jorma I. Virtanen

**Affiliations:** 1 Department of Dental and Oral Pathology, Lithuanian University of Health Sciences, Kaunas, Lithuania; 2 Department of Preventive and Pediatric Dentistry, Lithuanian University of Health Sciences, Kaunas, Lithuania; 3 Department of Clinical Dentistry, University of Bergen, Bergen, Norway; 4 Department of Community Dentistry, Institute of Dentistry, University of Turku, Turku, Finland

**Keywords:** traumatic dental injuries, treatment, complications, children and adolescents

## Abstract

**Objective** Traumatic dental injury (TDI) is a common dental concern among children worldwide. We performed a retrospective patient register study among children under 18 years to investigate TDIs with respect to causes, treatment, and complications.

**Materials and Methods** We collected information on TDIs from the original patient records of 407 child patients visiting dental clinic of Lithuanian University of Health Sciences, Kaunas, Lithuania. We analyzed all child patients’ ( *n* = 407) background, cause, type of TDI, treatment, complications, and time elapsed from injury to visit to the dentist.

**Statistical Analysis** The *χ* ^2^ -test, analysis of variance (ANOVA), and Kruskal–Wallis and Mann–Whitney tests served in the statistical analyses.

**Results** A total of 579 TDI cases occurred during 2010 to 2016. Lateral luxation (19.8%) and intrusion (14.8%) occurred more often in the primary than the permanent dentition ( *p* < 0.05). The most common cause of TDI was falling (56%). Avulsion occurred in approximately 10% of cases. Follow-up (44.5%) and tooth extraction (48.3%) were the most frequent treatments in the primary and splinting (25.3%) in the permanent teeth. Pulp necrosis was the most frequent complication in primary (92%) and permanent (54%) dentition. About 1% of the patients obtained dental care during the first hour after injury.

**Conclusion** The most frequent TDIs included lateral luxation in primary teeth and enamel-dentine fractures in permanent teeth. We observed a delay in patients obtaining emergency dental care.

## Introduction

Dental traumas are injuries to the teeth, periodontium, and surrounding soft tissues. They are quite common in dentistry, comprising 5% of all traumatic injuries in people seeking first aid and up to 17% of all bodily injuries among preschool children. 1

Children and adolescents experience mild or severe dental traumas from various causes, such as unsafe playing on playgrounds, accidents at schools, accidents in car crashes, or violence. 2 According to Andersson, 1 the prevalence of traumatic dental injuries (TDIs) in children and adolescents is approximately 20% and varies little. Petti et al 3 found that traumatic dental injuries occur in both primary and permanent dentitions, although the prevalence in primary dentition is higher. Prevalence differs with age and sex, with a global male-to-female ratio of 1.43, suggesting that men are more likely to develop TDI than are women. 3

TDI in primary dentition can affect the development of permanent teeth. 4 Damage and/or disturbances to permanent teeth and germs, depending on the mouth area affected, 5 can range from mild to severe. TDI in permanent teeth can cause permanent complications, such as pulp necrosis and internal and/or external root resorption, 6 and influence maxillofacial development. 7

In most cases, emergency care after TDI is needed to improve tooth prognosis and prevent complications. Dental avulsion, for example, is one of a few emergency situations in dentistry where urgent help is needed to save the tooth. 8 Even if an avulsed tooth is replanted immediately (within 5 minutes after TDI), the success rate may not be 100%. First aid should therefore be provided on site, ideally by medical personal, but also by parents, teachers, coaches, or capable available persons. 8 However, studies indicate that teachers and coaches lack adequate knowledge of appropriate behavior in emergency situations involving dental trauma. 9 10

Children with dental traumas are an important concern nowadays, not only for their possible negative outcomes and frequent common occurrence 11 but also because they can reduce quality of life. 12 13 In addition, people with untreated dental traumas more often experience chewing problems and difficulties with social interaction, such as excessive concern about what others think, avoiding smiling and laughing, and not talking to other children. 14

In Lithuania, general dentists in primary health care centers or specialists in hospitals are the main providers of first aid for children who suffer TDI. Typically, TDI patients first visit their primary health care center or a private clinic of their choosing. General dentists usually refer patients with complicated or severe TDIs or both to pediatric dental specialists in hospitals. Yet, research on TDI is scarce in Lithuania. Thus, our aim was to investigate the causes of traumatic dental injuries (TDIs), time elapsed from injury to first visit to the dentist, treatment method, and complications in children under 18 years.

## Materials and Methods

We conducted this retrospective patient register study at the Department of Preventive and Pediatric Dentistry, Lithuanian University of Health Sciences, Kaunas, Lithuania, in 2018. We analyzed the patient records of all patients under 18 years who visited the clinic due to dental trauma in the primary and/or permanent dentition between 2010 and 2016. The Bioethics Centre of the Lithuanian University of Health Sciences approved the study protocol (number BEC-OF-11). The patients’ parents were informed about the study and the anonymous use of their child’s dental records at the time they visited the clinic for dental care. All patients or their parents provided their written informed consent.

### Data Collection

We collected information on TDIs from the original paper records of 407 child patients. One researcher (A.A.) transferred the data from the patient records to a specifically designed SPSS template. We registered and categorized the information as follows: patients’ background information, cause and type of traumatic dental injury (TDI) according to Andreasen et al 15 criteria, traumatized teeth, and time elapsed from injury to first visit to the dentist. Radiographs were used during patients’ examination and treatment. Photographs were not routinely taken of patients with TDI.

Thereafter, we registered the treatment method and complications and categorized the registered data ( Table 1 ).

**Table 1:** The registered and categorized information of TDIs among the children and adolescents ( *n* = 407)

Category	Characteristics
Background information	Age (0–3, 4–8, and 9–17 years); gender (boys, girls); place of residence (urban, rural)
Cause of traumatic dental injury	Falling, cycling, playing, fighting, other
Traumatized tooth	The type of traumatized tooth in upper or lower jaw. Permanent premolars and molars were combined into two categories: upper posterior and lower posterior. Primary first and second molars were combined into two categories: upper posterior molars and lower posterior molars
Type of trauma according to Andreasen et al ^15^	Fractures: enamel infraction, enamel fracture, enamel-dentine fracture, enamel-dentine-pulp fracture, uncomplicated crown-root fracture (without pulp involvement), complicated crown-root fracture (with pulp involvement), root fracture, alveolar fracture. luxations: concussion, subluxation, extrusion, lateral luxation, intrusion, and avulsion
Time elapsed from injury to first visit to the dentist	Within an hour, 1–7 hours, the day after TDI, 2–6 days after TDI, 1 week or more after TDI
Treatment method	Restoration (GIC, composite), pulp capping (Ca(OH) _2_ ; MTA); pulpotomy; root canal treatment; tooth splinting; tooth extraction; and orthodontic extrusion of a traumatically intruded tooth
Complications	Marginal periodontitis, pulp necrosis, chronic periapical periodontitis, root canal obliteration, abscess formation, external root resorption, and internal root resorption
Abbreviations: TDI, traumatic dental injury; GIC, glass ionomer cement; MTA, mineral trioxide aggregate.	

### Statistical Analysis

We used IBM SPSS Statistics for Windows (version 22.0; Armonk, New York, United States) package to perform the statistical data analysis. Statistics served to describe the basic features of the data in the study, with the Chi-square test for determining relationships between categorical variables, and one-way analysis of variance (ANOVA) for comparing the means between groups. We also used the Kruskal–Wallis and Mann–Whitney tests, and *p* < 0.05 was considered statistically significant.

## Results

We analyzed the medical records of 407 TDI patients (62% boys and 38% girls) aged up to 18 years. Table 2 presents the demographic characteristics of the study participants. Of the 579 TDI cases, 281 (54.1%) involved permanent teeth and 238 (45.9%) involved primary teeth. The highest number of TDIs occurred among 0- to 3-year-old children ( *n* = 160; 39.3%), though the numbers declined with age (4- to 8-year-old children, *n* = 134, 32.9%; and 9- to 17-year-old children, *n* = 113, 27.8%).

**Table 2:** Background of the children and adolescents with TDIs ( *n* = 407)

	*n*	%
Age (y)		
0–3	160	39.3
4–8	134	32.9
9–17	113	27.8
Gender		
Boy	252	62
Girl	155	38
Place of residence		
Urban	309	76
Rural	98	24
Tooth type		
Primary teeth	238	45.9
Permanent teeth	281	54.1
Total	579	100
Abbreviation: TDI, traumatic dental injury.		

Table 3 shows the distribution of the various types and frequency of TDI in primary and permanent dentition. Lateral luxation (19.8%) and intrusion (14.8%) were diagnosed significantly ( *p* < 0.05) more often in primary than in permanent dentition (12.8 vs. 3.6%). Enamel-dentine fractures occurred significantly more often in permanent (33.5%) than in primary dentition (19.8%; *p* < 0.05). Avulsion occurred in 10% of permanent and 9.8% of primary teeth. Upper-central incisors were the most affected teeth in both dentitions (70.7% primary and 62.1% permanent), followed by upper-lateral incisors (13.8% primary and 26.6% permanent).

**Table 3:** TDIs (n, %) according to Andreasen et al 15 : classification and the injured tooth

TDI	Primary teeth *n* (%)	Permanent teeth *n* (%)
Infraction	0 (0)	2 (0.7)
Enamel-dentin fracture without pulp involvement	37 (19.8) ^a^	131 (33.5) ^a^
Enamel-dentine fracture with pulp involvement	24 (13.1)	43 (11)
Root fracture	10 (5.1)	20 (5)
Concussion	12 (6.3)	32 (8.2)
Subluxation	19 (10.1)	50 (12.8)
Extrusion	4 (2.1)	10 (2.5)
Intrusion	28 (14.8) ^a^	14 (3.6) ^a^
Lateral luxation	37 (19.8) ^a^	50 (12.8) ^a^
Avulsion	17 (8.9)	39 (10)
Injured tooth		
Upper central incisor	133 (70.7)	243 (62.1)
Upper lateral incisors	26 (13.8)	104 (26.6)
Upper canine	7 (3.7)	1 (0.3)
Upper posterior	4 (2.1)	2 (0.5)
Lower central incisor	8 (4.3)	30 (7.7)
Lower lateral incisor	5 (2.7)	11 (2.8)
Lower canine	3 (1.6)	0 (0)
Lower posterior	2 (1.1)	0 (0)
Abbreviation: TDI, traumatic dental injury. ^a^*p* < 0.05 between primary and permanent teeth.		

The number of TDIs varied across different age groups. Two age categories showed the highest number of dental traumas: 1- to 2-year-old children ( *n* = 130; 31.9%) and 7- to 9-year-old children ( *n* = 121; 29.7%). More girls ( *n* = 74) suffered dental trauma at a younger age (0–3 years) than at an older age (9–18 years; *n* = 32). TDIs in boys showed no variance between age groups.

**Fig. 1:**
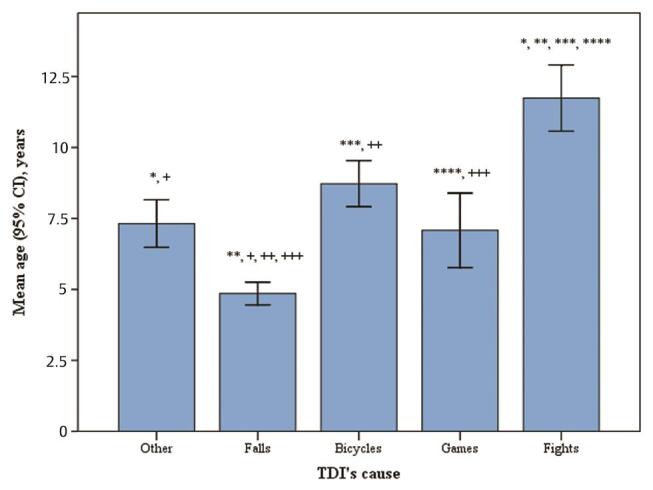
Patient’s age and cause of TDIs. CI, confidence interval; TDI, traumatic dental injury.

The most common cause of TDI was falling (56%), followed by riding a bicycle (10%), fighting (6%), and playing (5%; Fig. 1 ). Different causes dominated in different age groups: the younger children (mean age, 4.85 years, standard deviation [SD] = 3.36) experienced TDIs from falling, whereas fighting was the most common cause of TDI among the older children (mean age, 11.74 years, standard deviation (SD) = 2.94).

In our study, we observed a delay in obtaining care at a dental clinic. Less than 1% of the children came to the clinic during the first hour after injury, and about half of the patients came to the clinic within 1 to 7 hours after TDI. Only 3.9% of dental avulsion cases obtained dental emergency care during the first hour after injury. The time elapsed from dental trauma to obtaining emergency care at a dental clinic was shorter among older patients than among younger ones.

Table 4 shows treatment methods applied to the dental trauma in primary and permanent dentition. In 1.8% of cases involving avulsion of permanent teeth, the teeth were missing or unsuitable for replantation. Patient follow-up (44.5%) and tooth extraction (48.3%) were the most frequent treatments for TDI involving primary teeth. In permanent dentition, treatment of patients with TDI most often involved splinting of the traumatized tooth (25.3%), patient follow-up (22.5%), and temporary filling with glass ionomer cement (GIC) (21.4%).

**Table 4:** Treatment method (%) applied for TDIs in children and adolescents

Treatment	Primary teeth (%)	Permanent teeth (%)
Follow up/no treatment needed	44.5	22.5
Temporary restoration with a GIC	2.9	21.4
Composite restoration	–	7.5
Reattachment of fractured tooth fragment	0.4	1.8
Pulp capping with MTA/Ca(OH) _2_ and GIC/composite	–	9.7
Pulpotomy	–	0.7
Root canal treatment	3.4	3.6
Apexification with Ca(OH) _2_ /MTA	–	0.8
Splinting	0.4	25.3
Root canal treatment prior to replantation and splinting	–	1.1
Splinting and root canal treatment	–	1.1
Tooth extraction	48.3	2.1
Orthodontic extrusion of traumatically intruded tooth	–	0.4
Abbreviations: TDI, traumatic dental injury; GIC, glass ionomer cement; MTA, mineral trioxide aggregate.		

More complications occurred in the early period (<3 months after TDI) than later, but the difference failed to reach statistical significance. Complications related to pulp necrosis (pulp necrosis, periapical periodontitis, and abscess formation) were the most frequent complications in primary (92%) and permanent (54%) dentition ( Table 5 ). Enamel-dentin fractures without pulp involvement in primary and permanent dentitions, and dental avulsion in permanent dentition, caused complications more often than did other TDIs ( *p* < 0.05).

**Table 5:** TDI treatment complications in primary and permanent teeth among children and adolescents ( *n* = 407)

Complication	Primary teeth (%)	Permanent teeth (%)
Pulp necrosis, periapical periodontitis, and abscess	92	54
Internal, external root resorption, and root canal obliteration	4	24
Tooth loss	0.4	4
Abbreviation: TDI, traumatic dental injury.		

Avulsion occurred in 10% of permanent and 9.8% primary teeth. The avulsed tooth storage media is very important for the successful treatment of the avulsed tooth. Half of the patients seeking first aid care stored the traumatized tooth in dry media or water. Two-thirds (68%) of the avulsed teeth developed complications; most complications (57.9%) occurred during the first 3 months after treatment (not shown in the tables).

Significantly, more TDIs occurred in summertime than in winter or spring ( *p* < 0.05). The number of TDIs showed no significant differences between weekdays.

## Discussion

This comprehensive register-based study analyzed the dental records of all child patients under 18 years visiting the University Clinic due to dental trauma over a 7-year period. TDIs occurred most frequently among boys, though a significantly higher number of TDIs among girls occurred in the youngest age group (0–3 years old). We observed a significant delay in patients obtaining emergency dental care.

Traumatic dental injury (TDI) is a common dental concern among children of various ages worldwide; its prevalence, however, unlike that of dental caries, dental development, or periodontium diseases varies depending on social and cultural factors. In our study, the number of dental traumas peaked among toddlers (1–2 years old) and 7- to 9-year-old school children. These findings are in line with the results of similar studies, 16 17 though other studies have reported TDI peaks in older school children (8–10 years) 18 19 20 and found no similarly high frequency of TDIs in toddlers. 21 In our study, most TDIs (87.7%) occurred in children under 10 years, while only 13.3% of TDIs occurred in children over 10 years.

In our study, boys (62%) suffered dental trauma more often than did girls (38%), a finding reported earlier in other studies. 17 18 20 21 In addition, 9- to 18-year-old boys were significantly more likely than girls to experience TDI, a tendency attributable to behavioral factors and expressions of emotion related to the different sexes, 22 such as boys participate in contact sports more often than girls. A new and interesting finding in this study was that in the toddler group, girls suffered dental trauma more often than boys of the same age, though the reason for this phenomenon in our study remained unclear.

The most common cause of TDI was falls which is in line with other research findings. 16 17 20 23 This study identified riding a bicycle, fighting, and playing as causes of TDI; comparable studies have reported similar findings. 16 17 Since various sport activities among Lithuanian children and teenagers are increasingly popular, these have likely attributed to the causes of TDIs. The most frequently injured teeth in our study were the upper-central incisors in both dentitions, a finding in line with earlier studies. 16 17 23

In our study, we observed a delay in patients obtaining emergency dental care. Less than 1% of children obtained emergency care within the first hour after trauma, and about half of the patients did so within 1 to 7 hours after injury. Unfortunately, this figure, as reported elsewhere, is unexceptional. 16 20 23 Immediate emergency care is crucial for the successful treatment of dental trauma; the time lapse between injury and first aid affects both tooth survival prognosis and treatment outcome. 24 This is especially important in avulsion cases, 23 25 yet our findings showed that patients obtained emergency care during the first hour after injury in only 4% of dental avulsion cases.

One reason for the observed delay in patients obtaining emergency care could be that, at the time of trauma, the immediate help of health personnel is unavailable. Those available to help are usually parents and teachers, but studies have shown that their level of knowledge related to dental emergency care and the importance of visiting a dentist immediately after TDI are limited. 9 A systematic review and meta-analysis by Tewari et al showed that school teachers generally exhibited low self-belief and knowledge level of TDIs. 26

We used Andreasen et al 15 criteria to classify the TDIs. Many authors of TDI studies widely recognize and use this classification system. 16 17 Lopez et al, 27 for instance, used this classification in their systematic review and meta-analysis of the impact of TDIs on the quality of life of children and adolescents. Several studies have shown that luxation injuries occur more often in the primary dentition, while fracture injuries are more likely to occur in the permanent teeth. 16 17 20 23 28 Our study showed that lateral luxation and intrusion occurred more often in the primary dentition than in the permanent dentition, while enamel-dentine fractures occurred more frequently in the permanent dentition than in the primary dentition.

Most TDIs (87.7%) occurred in children younger than 10 years. The age period from 7 to 10 years is especially vulnerable because at that age, the root development of the permanent incisors is still incomplete, and delayed emergency care after TDI will increase the risk for complications, making vital tooth survival, and/or good treatment prognosis less predictable. The most frequent treatment method for the permanent dentition was splinting (25.3%), followed by temporary restoration of the fractured tooth with GIC. This finding contrasts with some previous findings where, in cases of delayed referral to a clinic, root canal treatment was the most common treatment method. 20 In our study, the most common (48.3%) treatment method among patients with TDI in the primary dentition was tooth extraction, possibly due to efforts to avoid complicated operative treatment and to save the germs of permanent teeth in cases of serious TDIs involving the primary teeth. Other reports identified follow-up as the most frequent procedure after TDI, 20 21 whereas in our study, follow-up took place in less than half of the cases.

Dental avulsion occurred in 10% of all TDIs. This is a higher percentage than in other studies, 8 16 17 though a tertiary teaching hospital in Australia reported a similar finding, 23 possibly because dentists working in primary care refer only the most severe cases to hospital care. In our study, patients with avulsion went directly to the Department of Preventive and Pediatric Dentistry rather than through primary care. A recent survey among Lithuanian general dental practitioners showed that they lacked sufficient knowledge of dental trauma. 29 Researchers have also observed similar findings in other countries as demonstrated in the global study of dental professionals’ knowledge by Tewari et al, 30 possibly because general dental practitioners may possess only moderate knowledge of TDI, whereas specialists in endodontics and pediatric dentistry possess greater knowledge, 31 medical doctors also often lack sufficient knowledge of dental trauma. 32

The most frequent complications were pulp necrosis, periapical periodontitis, and abscess formation, occurring in 94% of primary and 54% of permanent teeth. This may at least partly result from the delay in obtaining emergency care and the fact that most TDIs occurred in children under 10 years. The age period from 7 to 10 years is especially vulnerable because at that age, the root development of the permanent incisors is still incomplete.

Population-based epidemiological studies on the prevalence and severity of TDI, however, are lacking. 33 Our comprehensive study was based on a sample of all TDI patients at the Department for Preventive and Pediatric Dentistry, Lithuanian University of Health Sciences, from 2010 to 2016 and included a relatively high number of dental trauma cases. The Lithuanian University of Health Sciences Hospital is the second largest in Lithuania, and patients from all regions of the country seek care there. The data do not, however, include all emergency TDIs from the whole country. On the other hand, use of the criteria by Andreasen et al 15 to classify the TDIs enables comparison of our findings to those of other studies from different countries. Future studies on the prevalence and severity of TDI, with emphasis on the reasons for delays in obtaining emergency care, are necessary.

## Conclusion

The most common cause of TDI was falling, followed by riding a bicycle. The most frequent TDIs involved lateral luxation and enamel-dentine fractures in the permanent teeth. Complications related to pulp necrosis were the most frequent complications in primary and permanent dentition. Our study found a considerable delay in patients obtaining dental emergency care, suggesting a lack of knowledge among patients, parents, and teachers of its importance immediately after TDI. We therefore recommend more community-level educational programs related to TDIs.
